# Increased anterior cingulate cortex response precedes behavioural adaptation in anorexia nervosa

**DOI:** 10.1038/srep42066

**Published:** 2017-02-13

**Authors:** Daniel Geisler, Franziska Ritschel, Joseph A. King, Fabio Bernardoni, Maria Seidel, Ilka Boehm, Franziska Runge, Thomas Goschke, Veit Roessner, Michael N. Smolka, Stefan Ehrlich

**Affiliations:** 1 Division of Psychological and Social Medicine and Developmental Neuroscience, Faculty of Medicine, Technische Universität Dresden, Dresden, Germany; 2 Eating Disorder Treatment and Research Center, Department of Child and Adolescent Psychiatry, Faculty of Medicine, Technische Universität Dresden, Dresden, Germany; 3 Department of Psychology, Institute of General Psychology, Biopsychology and Methods of Psychology, Technische Universität Dresden, Dresden, Germany; 4 Department of Psychiatry and Neuroimaging, Technische Universität Dresden, Dresden, Germany

## Abstract

Patients with anorexia nervosa (AN) are characterised by increased self-control, cognitive rigidity and impairments in set-shifting, but the underlying neural mechanisms are poorly understood. Here we used functional magnetic resonance imaging (fMRI) to elucidate the neural correlates of behavioural adaptation to changes in reward contingencies in young acutely ill AN patients. Thirty-six adolescent/young adult, non-chronic female AN patients and 36 age-matched healthy females completed a well-established probabilistic reversal learning task during fMRI. We analysed hemodynamic responses in empirically-defined regions of interest during positive feedback and negative feedback not followed/followed by behavioural adaptation and conducted functional connectivity analyses. Although overall task performance was comparable between groups, AN showed increased shifting after receiving negative feedback (lose-shift behaviour) and altered dorsal anterior cingulate cortex (dACC) responses as a function of feedback. Specifically, patients had increased dACC responses (which correlated with perfectionism) and task-related coupling with amygdala preceding behavioural adaption. Given the generally preserved task performance in young AN, elevated dACC responses specifically during behavioural adaption is suggestive of increased monitoring for the need to adjust performance strategies. Higher dACC-amygdala coupling and increased adaptation after negative feedback underlines this interpretation and could be related to intolerance of uncertainty which has been suggested for AN.

Anorexia nervosa (AN) is an eating disorder that is characterised by preoccupations with weight and shape, an intensive fear of weight gain, and a fierce pursuit of weight loss, mostly by self-starvation. Clinical observations suggest that AN patients have a strong drive to adhere to strict behavioural routines and are prone to perseverative, obsessive, and rigid thinking styles. Accordingly, personality characteristics like perfectionism, low impulsivity, intolerance of uncertainty and high harm avoidance are common and often seen already premorbidly in individuals with AN^1^,^2^,^3^. These cognitive styles and behavioural schemata may have adverse effects on AN patients’ decision making capacity, e.g. they may interfere with the ability to learn from experience^4^.

Indeed, data from behavioural experiments suggests reduced cognitive flexibility in acute AN, i.e. impaired disengagement from previously relevant rules in order to learn new ones^5^,^6^. Most studies have employed experimental paradigms, which test the ability to switch back and forth between multiple tasks or mental sets^7^. Impairments in the ability to switch between multiple tasks or mental sets, i.e. “set-shifting” has even been suggested as an endophenotype for AN^5^ and may partially explain the maintenance of symptoms and persistence to change during psychotherapeutic interventions^8^. However, while impaired set-shifting has also been reported in recovered AN patients^5^, more recent studies indicate that adolescent patients with acute AN are largely unaffected^9^,^10^,^11^,^12^,^13^, suggesting that set-shifting impairments increase as a consequence of the illness.

The behavioural impairments in cognitive flexibility described above, together with the striking ability of AN patients to restrict their diet to only a few hundred calories per day, have fostered the notion that AN may be viewed as a model disorder for abnormally increased self-control. Further support has come from recent functional magnetic resonance imaging (fMRI) studies showing abnormal neural responses in brain regions implicated in cognitive control including subregions of the anterior cingulate cortex (ACC) and the dorsolateral prefrontal cortices (DLPFC)^14^ during simple target detection^15^, reward processing^16^,^17^,^18^, response inhibition^19^,^20^,^21^, set-shifting^22^ and working memory^23^. Similarly, we and others have found altered functional connectivity in acute AN patients in the ACC^24^,^25^ and the fronto-parietal network^25^,^26^ at rest, which may, again, indicate increased control over ‘bottom up’ processes such as reward and emotions^27^.

Research in healthy cohorts demonstrates strong relations between cognitive control, eating, and weight. For example, deficient response inhibition has been associated with weight gain^28^, Another study suggested that the orbitofrontal cortex (OFC) integrates competing goal values when choosing between healthy versus tasty food and that this process is modulated by the DLPFC – one of the core areas of the cognitive control system^29^. On a related note, patients with AN willingly endure cold or subject themselves to torturous exercise regimes – all with the goal to burn calories and lose more weight. In line with this seemingly extraordinary will power, patients were reported to have a tendency to delay immediate but smaller for larger rewards in the future (decreased delay discounting^30^) – although the latter finding has not been replicated using other delay discounting tasks^31^,^32^,^33^.

Taken together, these seemingly contradictory findings and observations leave us with a riddle. On the one hand, behavioural data from set-shifting experiments in adult patients suggest impaired task-set updating and difficulties with goal-directed behaviour. On the other hand, neuroimaging and decision making research as well as clinical observations are suggestive of excessive cognitive control in AN.

In order to gain a deeper understanding of decision making and cognitive control in AN, we studied probabilistic reversal learning instead of (overt) set-shifting and focused on young non-chronic patients with acute AN. The latter has the advantage of avoiding secondary effects of pro-longed undernutrition on general cognitive abilities^10^. Probabilistic reversal learning (PRL) requires the adaptation of behaviour according to covert changes in stimulus-reward contingencies^34^, a capacity relevant in day-to-day life, particularly in complex social situations^35^, in which AN patients often struggle. In detail, during PRL participants must learn to implicitly detect (changes in) stimulus-reward contingencies and subsequently respond flexibly to the opposite, previously disadvantageous stimulus. To mirror real-life situations, PRL includes probabilistic feedback, i.e. participants may experience a negative outcome despite a correct choice and vice versa. This task has a high ecological validity and allows differentiating aspects of performance monitoring and value-based decision making (such adapting behaviour after negative feedback) in a dynamic environment.

Data from animal models, adolescent and adult healthy individuals as well as patients following neurosurgical resection have implicated a number of well-characterised brain regions in reversal learning^36^,^37^,^38^,^39^,^40^,^41^,^42^. The ventral striatum is thought to represent the value assigned to the different possible choices and reflect the difference between the expected value and the actual outcome of an action^43^,^44^,^45^. The ventromedial prefrontal cortex mediates the learning and retrieval of (subjective) value information that guides decision-making^46^,^47^. Finally, the dorsal division of the anterior cingulate cortex (dACC) is crucial to adaptive goal-directed behaviour, monitoring the quality of performance vis-à-vis changing environmental demands, detecting erroneous or error-prone actions, and integrating reinforcement history^48^,^49^,^50^. During PRL, the dACC is believed to signal the need for compensatory adjustments that drive subsequent changes in behaviour^51^,^52^. The ACC (including dACC) is also considered one of the key brain region implicated in eating disorder pathology^53^,^54^.

Here we aimed to investigate PRL and its neural correlates in young non-chronic acute AN patients using functional magnetic resonance imaging and a stringent study design (to exclude possibly confounding effects of medication, realimentation or age). Although it remains an empirical question whether impairments in behavioural flexibility would be apparent in the context of PRL and a young cohort, we specifically hypothesised to find correlates of increased performance monitoring (especially involving the dACC) in the patient group.

## Results

### Sample Characteristics and Behavioural Data

There were no differences in age, IQ, or handedness score between the pairwise matched groups of AN and HC. However, as expected AN had a significantly lower BMI and lower minimal lifetime BMI. Furthermore, AN had significantly higher eating disorder symptom and depression scores (Table 1).

While there were no group differences in total win, persistence, occurrence of win-shift or number of contingency reversals, occurrence of lose-shift was increased in AN relative to HC (Table 1).

### Imaging Data

**Figure 1:**
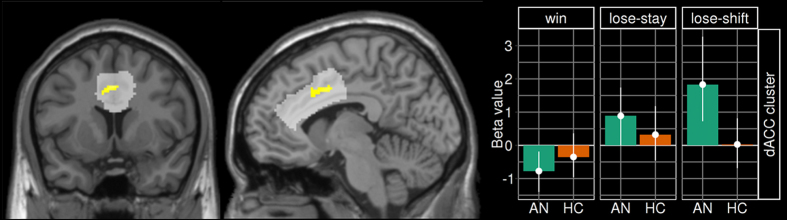
Group-Feedback interaction. Left: Brain map depicting the interaction effect with peak at x = −11, y = 6, z = 36 [F(2,140) = 9.5, p_FWE-SVC_ <0.001]. ACC ROI is highlighted in light grey. Right: Extracted beta values of the dACC cluster region across groups and feedback-condition depicted as a bar chart (means with 95% CI).

Results of the mixed model including a three-level within-subject variable (feedback) and a binary between-subject variable (group) are depicted in Fig. 1 and SI Figure 2.1. Confirming Hampton *et al*.^44^, we found a significant main effect of feedback across all participants in the following *a priori* ROIs: ACC, VS, left DLPFC (lDLPFC), and right DLPFC (rDLPFC) (see SI Table 2.2). No general group differences (main effects) reached significance in any ROIs. More importantly, a significant feedback x group interaction was revealed in the dACC (Fig. 1). When using extracted beta-values and a mixed model post-hoc tests of this interaction [F(2,140) = 7.3, p < 0.001] revealed an increased neural response to lose-shift (z = −2.89, p = 0.011) in AN.

Analyses of correlations between the pattern of hemodynamic activity in the dACC cluster during lose-shift showed no relationships with age, IQ, BMI-SDS, minimal lifetime BMI, BDI-II, or EDI-2 total score in either of the groups (AN: all |r| < 0.13, all p > 0.49; HC: all |r| < 0.23, all p > 0.19). However, the EDI subscale perfectionism was positively correlated with the dACC responses during lose-shift in AN (r = 0.356, p = 0.039), but not in HC (r = −0.097, p = 0.577).

Analyses of correlations between neural responses in the dACC cluster during lose-shift and the behavioural occurrence of lose-shift revealed a positive relationship in HC (r = 0.379, p = 0.023).

### Psycho-physiological Interaction

**Figure 2:**
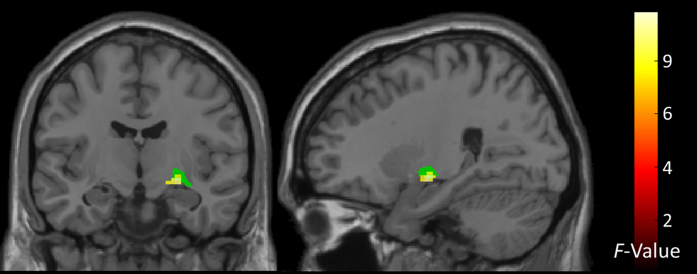
gPPI results. Statistical maps (whole-brain analysis, p < 0.001 uncorrected, cluster extent k> = 30) showing regions of group differences in functional connectivity across task conditions (warm colours). Global peak at x = 25, y = −10, z = −8 [F(2,140) = 9.9, p < 0.001]. Seventy-seven percent of the cluster overlaps with the right centromedial amygdala (coloured in green) defined by the Jülich probabilistic atlas^104^.

Having discovered a group x feedback interaction in the dACC, we asked whether the degree of feedback-dependent neural responses in this region functionally covaried with that in other regions of the brain. In a whole brain analysis, one region showed a group difference in functional connectivity with the dACC across feedback conditions (group x feedback interaction): the right amygdala (Fig. 2). Comparison of extracted beta-values suggested that functional coupling between these two regions was highest in AN during the lose-shift behaviour but reduced during lose-stay (SI Figure 2.2).

## Discussion

The current study used fMRI during performance of a probabilistic reversal learning task to investigate the neural correlates of cognitive flexibility in response to changing reward contingencies in acute AN patients. Although task performance in AN was generally unimpaired and highly comparable to that observed in HC, patients showed increased shifting behaviour specifically after receiving negative feedback (lose-shift). This differential sensitivity to feedback between the AN and HC groups was mirrored in feedback-dependent neural responses in the dACC. Specifically, in AN increased neural response in the dACC on trials with negative feedback were followed by behavioural adaptation. Furthermore, group differences in feedback-dependent changes in functional connectivity between this dACC cluster and right amygdala were found. Together, as argued below, these findings are consistent with the notion of elevated performance monitoring in AN.

The dACC has been consistently implicated in monitoring performance quality and integrating reinforcement history for the need for adjustments in cognitive control^14^,^50^. For example, much evidence demonstrates that the dACC is responsive to conflict and negative feedback, like errors in task performance or unexpected events^48^,^55^,^56^. A recent formal model of cognitive control, the Expected Value of Control framework (EVC)^57^ posits that the strength of a control signal that drives adjustments in cognitive control – assumed to be provided by dACC – depends on cost-benefit calculations (the difference of the expected value and the invested effort to provide a control signal required to solve a given task). Increased dACC activity after negative feedback in AN may indicate that AN assign a larger weight to negative feedback, which should lead to a relative increase of the expected value of recruiting control in order to maximize reward. This could explain the increased lose-shift behaviour, if one assumes that such switches require control to overcome the perseverative tendency to continue with the previously successful response strategy.

In AN, the same region of the dACC identified in the current study and neighbouring regions of the ACC have been implicated in resting state studies reporting aberrant functional connectivity with the inferior frontal gyrus^58^ and both retrosplenial cortex and precuneus^25^. A recent resting state study in adolescents found decreased temporal correlation between activity in the executive control network and a slightly more rostral ACC region; suggesting such alteration may represent an early trait-marker for the disorder^24^. Aberrant BOLD responses of the ACC in AN have also been found in task-based studies. For example, one study focusing on inhibitory control found increased activation in rostral ACC during a go/no-go-task in AN^20^. Another study reported elevated activation in the same dACC region identified in the current study during an emotional stroop task^59^, while another found increased mid-cingulate activity during pain processing in AN^60^. These findings are all compatible with the notion of increased action monitoring in AN (but see Wierenga *et al*.^21^). This notion is further supported by the fact that in our study higher values of self-reported perfectionism in AN were associated with higher dACC activation in response to lose-shift behaviour.

Furthermore, our interpretation is in line with additional studies indicating increased engagement of cognitive control brain circuitry (predominantly DLPFC) in AN during incentive processing and motivation-related tasks. These tasks predominantly recruit limbic structures when performed by healthy individuals^16^,^17^,^33^,^61^,^62^. For example, we recently found DLPFC activity during performance of a monetary incentive delay task to show stronger functional coupling in AN with mOFC^16^, a brain region thought to be crucial for the representation of subjective value on a common scale^63^, which may suggest increased control of valuation processes^29^.

Given the probabilistic schedule of reward contingencies in our task, the increased number of lose-shift behaviour in patients may reflect increased uncertainty. In line with these findings, a recent behavioural study using a similar task in chronic adult AN patients also reported generally preserved performance but increased shifting after punishment^64^. Increased performance monitoring by the dACC may be related to an intolerance for uncertainty and need for control, which has been suggested as a basis for a psychological model of AN^3^. Of note, increased dACC activation in the current study in HC was correlated with a higher occurrence of lose-shift behaviour. This observation suggests that HCs that are similar to AN in terms of dACC activation (increased performance monitoring) are also more likely to show explorative behaviour. Functional connectivity between amygdala and the rostral “affective” subdivision of the ACC has been described to be involved in identification of emotional relevance of stimuli and production of adequate behaviour in response to their motivational significance^65^. The observed increase in functional connectivity between dACC and amygdala in AN upon lose-shift might be interpreted in a similar way, that is, elevated amygdala-dACC coupling may potentially reflect uncertainty about the expected outcome of the previously chosen option incurring a change in behaviour (exploration of alternative options).

More evidence for disorder-related increased performance monitoring and intolerance of uncertainty has been repeatedly described in patients with obsessive-compulsive disorders (OCD)^66^. Patients with AN, are often found to have comorbid OCD and/or share specific core symptoms like perfectionism, demand for certainty, and compulsivity^67^,^68^,^69^. OCD patients show elevated uncertainty regarding action performance and decision making resulting in compulsive behaviours to reduce uncertainty^70^. Similar to our findings, dACC activity was found to be increased during performance monitoring in OCD patients^71^,^72^,^73^. In line with the common interpretation of ‘stronger recruitment of proactive control’ even at rest, OCD patients seem to show increased functional connectivity of the fronto-parietal attention/executive network (in particular of the dACC), which was also correlated with OCD symptoms^74^. This finding resembles recently published resting state connectivity data in acute AN^24^,^26^.

Our findings are also in line with a study by Evers and colleagues^75^, who employed acute tryptophan depletion as an experimental method to lower brain 5-HT availability during a PRL paradigm. Following depletion, they found unchanged behavioural performance but increased neural responses specifically in the dorsomedial prefrontal cortex after negative feedback incurred a change in behaviour. These results as well as evidence for a role of 5-HT in the processing of aversive signals^76^,^77^ implicate 5-HT in processes such as the detection/monitoring of errors and/or conflict. Low 5-HT states may thus increase sensitivity to negative feedback (enhanced processing of error feedback leading to enhanced response conflict), which we detect as increased BOLD signal in the dACC. Interestingly, a number of studies have shown reduced 5-HT activity in AN, which are thought to be secondary to a diet-induced reduction of tryptophan, the precursor amino acid of 5-HT^78^,^79^,^80^,^81^,^82^,^83^,^84^. Of note, a PET study found a positive correlation between reduced 5-HT_2A_ receptor activity, particularly in cingulate regions, and harm avoidance/fear of uncertainty in AN^85^.

However, there is another possible explanation for the findings of our current study. Adult AN patients are known for their reduced cognitive flexibility, rigidity, and impaired set shifting ability^5^,^6^,^86^, which is not found in children and adolescents suffering from AN compared to HC^87^. Our comparatively young, non-chronic sample shows equal overall task performance as controls (e.g. total win). Therefore, one could also assume that impairments regarding cognitive flexibility are compensated in young AN by higher recruitment of neuronal resources in the corresponding control regions. Increasing intensity of control regions of the brain is described by former studies to improve performance^57^. The elevated dACC activation in AN, displaying increased conflict monitoring and cognitive control, but equal task performance of both groups, may be seen as supportive evidence for this inefficiency hypotheses, previously described for individuals suffering from depression^88^ and schizophrenia^89^.

The following limitations of our study need to be considered. First, developmental effects due to the young age of our sample cannot be excluded. However, we closely matched for age to minimize possible developmental effects. It is important to note that although accounting for potential age effects did not influence the main results, our observations are limited to the investigated age range and may not apply to older, more chronic AN samples. Further, we might only capture a subgroup of individuals with some awareness of being ill as we recruit patients who seek psychiatric care. Second, severe undernutrition may lead to (pseudo-) atrophic grey matter changes^90^. Studies of brain function in acutely underweight AN may potentially be biased by the effects of (pseudo−) atrophic brain changes due to undernutrition^91^,^92^. Although exploratory analysis of relationships between cortical thickness measured in the identified region of the dACC and lose-shift activity in the same region revealed no significant correlations (see SI 1.9 and 2.1), future fMRI studies in acAN should also take the possibility of this confound into consideration. However, to avoid problems with registration of our functional data to a common coordinate system we have estimated a group-specific template during our image preprocessing (DARTEL)^93^. Third, it is difficult to disentangle whether functional brain abnormalities are cause or consequence of pathological eating. To ensure if our findings are state or trait markers of AN more studies in long-term recovered AN patients are needed. An important strength of our study is the large homogenous, unmedicated sample of young acute AN patients who have had a short duration of illness. Furthermore, we excluded effects of satiation status and circadian rhythm by scanning all participants in the morning after an overnight fast.

In conclusion, we found that although acutely ill AN patients show generally similar performance during PRL relative to HCs, they displayed increased behavioural shifting specifically after receiving negative feedback. This behavioural pattern was accompanied by increased dACC response in the AN group, which correlated with self-reported perfectionism. These findings are similar to those in OCD patients and support the notion that AN may also constitute a model disorder of abnormally elevated performance monitoring and cognitive control^27^. According to this view, hyperfunctioning of dACC might serve to recruit additional cognitive resources needed to adapt behaviour when goals (e.g. calorie restriction; skinny body) are in jeopardy. Higher dACC-amygdala coupling and increased lose-shift behaviour underlines this interpretation and could be related to an intolerance of uncertainty, which has been reported for AN^3^,^64^.

## Methods

### Participants

The sample consisted of 36 AN and 36 pairwise matched female HC (12–24 years old). AN participants underwent fMRI within 96 hours after beginning behaviourally oriented nutritional rehabilitation programs (see SI 1.1). For all participants, current and/or past diagnoses of eating disorders were diagnosed using a semi-structured research interview and the well-validated expert version of the Structured Interview for Anorexia and Bulimia Nervosa for DSM-IV [SIAB-EX^94^; Supplementary Information (SI) 1.2]. AN participants had to have a body mass index (BMI) below the 10^th^ age-percentile (if age <15.5 years) or a BMI below 17.5 kg/m^2^ (if age ≥ 15.5 years) and no recent weight gain.

HC participants had to be of normal weight (BMI ≥ 10^th^ age percentile if younger than 18 years or ≥ 18.5 kg/m^2^ if 18 years or older), eumenorrhoeic, and without any history of psychiatric illness (see SI 1.1). We applied several additional exclusion criteria for each group– most importantly a history of bulimia nervosa or “regular” binge eating, psychotropic medications within 4 weeks prior to the study, substance abuse, and neurologic or medical conditions (see SI 1.2).

In the AN group 6% of the participants had associated psychiatric comorbidity (6% depressive disorders, 1% obsessive-compulsive disorder, and 1% anxiety disorder).

This study was conducted in concordance with the guidelines laid down in the Declaration of Helsinki and was approved by the ethical committee of the TU Dresden. All participants (and their guardians if underage) gave written informed consent.

### Clinical Measures

To complement the information obtained with the clinical interviews, eating disorder-specific psychopathology was assessed with the German version of the Eating Disorders Inventory (EDI-2)^95^. Here we focused on the EDI-2 total score and the perfectionism subscale. Depressive symptoms were explored using the German version of the Beck Depression Inventory (BDI-II)^96^.

Intelligence quotient (IQ) was measured with short versions of the German adaptation of the Wechsler Adult Intelligence Scale (WIE; if age ≥16 years)^97^ or the Wechsler Intelligence Scale for Children (HAWIK; if age <15 years)^98^.

We used the BMI standard deviation score (BMI-SDS) instead of BMI for statistical analysis. For more details on psychiatric and psychological assessments see SI 1.2.

### Experimental paradigm

We used a PRL task adapted from Hampton *et al*.^44^. This decision-making task includes probabilistic positive and negative monetary feedback and contingency changes according to a learning criterion^40^.

**Figure 3:**
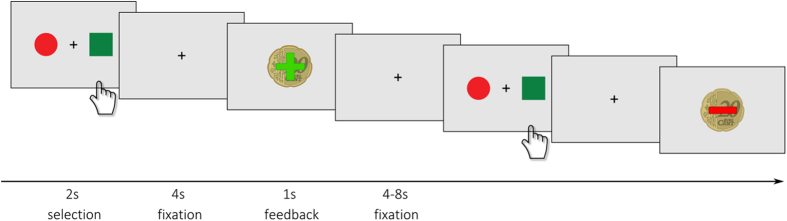
Experimental design. First, two abstract stimuli are presented for up to 2 s. After the participant selected one stimulus by left or right button press a fixation cross was presented for 4 s. Finally, positive or negative feedback (monetary reward or loss) was displayed for 1 s followed by a jittered inter-trial interval (fixation cross) for 4 to 8 s.

The task performed in the scanner consisted of 120 trials (total duration of ca. 26 min). In each trial, a coloured circle and a coloured square were presented on the left and right side of a screen (spatial position randomised; Fig. 3). Subjects were asked to choose one of the two stimuli by pressing the left or right button within 2 seconds after presentation. In 80% of the cases the choice of the implicitly designated ‘correct’ or ‘incorrect’ stimuli led to a positive feedback (+20 cents) or a negative feedback (−20 cents), respectively. In the remaining 20% a probabilistic error occurs, which means choosing the ‘correct’ stimulus led to a negative feedback and vice versa. With a probability of 25% the contingency reversed (change of the ‘correct’ figure to the previously ‘wrong’ figure) after at least four consecutive correct decisions, triggering a behavioural adaptation in the following trials. The total win was paid at the end of the session.

Before entering the scanner, participants performed a training run to become acquainted with probabilistic errors (see SI 1.3).

### Behavioural Data Analysis

The following behavioural performance measures were considered: total accumulated monetary reward (total win a participant achieved at the end of the experiment), occurrence of negative feedback that incurred a change in behaviour (lose-shift), occurrence of behavioural shift after receiving positive feedback (win-shift), number of contingency reversal (after the learning criterion was achieved) and persistence which is the average number of reversal errors after a contingency switch until the subsequent behavioural shift occurs (where a reversal error is defined as an incorrect response in consequence of applying the previously learned correct response). For information regarding quality control of behavioural data please refer to SI 1.4.

Group differences in the behavioural performance measures were examined using ANCOVA controlling for IQ (as in previous reports^64^, see SI 1.4).

### MRI Data acquisition

Images were acquired between 8 and 9 am in the morning after an overnight fast using standard sequences with a 3T whole-body MRI scanner (TRIO; Siemens, Erlangen, Germany).

The functional images were acquired by using a gradient-echo T2*-weighted echo planar imaging (EPI) with the following parameters: number of volumes = 656, number of slices = 42, repetition time = 2410 ms.

The T1-weighted structural brain scans were acquired with a rapid acquisition gradient echo (MP-RAGE) sequence with the following parameters: number of slices = 176, repetition time = 1900 ms, slice thickness of 1 mm. For more details see SI 1.5.

### MRI Data Preprocessing

Functional and structural images were processed using SPM8 toolbox (http://www.fil.ion.ucl.ac.uk/spm/) within the Nipype framework^99^. A DARTEL template was created using structural images from all 72 subject, namely HC and AN, of this study^93^. This ensures that the brain template represents the average brain of the study’s participants and prevents a potential group bias^100^.

The functional images were corrected for temporal slice-timing and motion simultaneously using realign4D^101^, which is an improved realignment algorithm that accounts for the combination of head motion during scanning and staggered slice acquisition in order to reduce the spatio-temporal distortion. The six realignment parameters, describing the rigid-body movement (x, y, z, pitch, roll, yaw), were saved and later used as nuisance covariates to account for the variance due to motion. The EPI volumes were coregistered to the subject’s structural brain image. This was followed by the normalization to MNI space using the DARTEL template and corresponding flow field. The resulting data were smoothed with an isotropic 8 mm FWHM Gaussian kernel.

We evaluated the quality of the fMRI data by manual inspection and using artefact detection tools (ART)^102^. Volumes that exceed a brightness intensity threshold of three standard deviations or a threshold of 2 mm normalised movement in any direction were classified as outliers. AN patients had numerically less outliers (number of motion-outliers: mean AN 7.8 ± 12.8 SD, mean HC 14.4 ± 23.1 SD; number of intensity-outliers: mean AN 9.1 ± 5.1 SD, mean HC 9.2 ± 5.2 SD), but the groups did not differ significantly in this measure (motion-outlier: t(70) = −1.52; p = 0.13; intensity-outlier: t(70) = −0.115; p = 0.91). The indices of the outlier volumes were saved and later used to create nuisance covariates in the 1st level GLMs.

### MRI Data Analysis

On the 1st level for every participant a General Linear Model (GLM) was fitted to model the brain activation during three feedback conditions (see Javadi *et al*.^40^): (i) win (which includes win-shift since these events were too rare to be modelled), (ii) the absence of a behavioural shift after receiving negative feedback (lose-stay), and (iii) behavioural shift after receiving negative feedback (lose-shift). Additional regressors included the event of stimulus presentation, six realignment parameters as well as one regressor for each motion or intensity outlier volume.

On the 2nd level a linear mixed model including a three-level within-subject variable (feedback: win, lose-stay, lose-shift) and a binary between subject variable (group: AN, HC) was estimated using GLM_flex (SI 1.5).

We generated contrasts to explore the main effect of group, main effect of feedback type and the effect of the interaction between group and feedback. We constrained our search space to the following atlas-based brain regions: three task-related regions of interest [ROIs; ventromedial prefrontal cortex (vmPFC), ventral striatum (VS), anterior cingulate cortex (ACC)] and two AN-related cognitive control ROIs [left and right dorsolateral prefrontal cortex (DLPFC)]. See highlighted areas in SI Figure 1.1 and SI 1.6 for more details.

All results were corrected for multiple comparisons based on small volume correction (SVC) with an α-level of 0.01. A cluster was considered as significant if its size exceeded the ROI-specific minimal cluster size (for ACC k = 97, for lDLPFC k = 99, for rDLPFC k = 97, for VS k = 34, and for vmPFC k = 32, see SI 1.6 for details).

To extract feedback-specific weighted averages (first eigenvariates) of the beta values from all voxels belonging to significant clusters, we used the MarsBaR toolbox for SPM. To investigate the direction of changes in BOLD responses, extracted beta values were subjected to a linear mixed model (see SI 1.7).

As follow-up analysis of functional connectivity, we used the generalised psychophysiological interaction approach (gPPI)^103^ to assess whether the effect of feedback (psychological factors: win, lose-stay, lose-shift) is significantly modulated by the activity of the seed region (physiological factor). Based on the findings in our main analysis (see results section), the seed was defined by a spherical region in the dACC (centred at peak MNI coordinates *x, y, z* = −11, 6, 36; radius 10 mm). For more precise information on analysis process see SI 1.8.

Associations between neural activity or connectivity and clinical or behavioural performance measures were carried out using SPSS. Given our interest in flexible decision making in AN, we focused on overall eating disorder symptoms (EDI-2 total score) and perfectionism (EDI2 subscale perfectionism). In order to guard against possible confounding influences, we also explored associations with depressive symptoms as well as age, IQ, BMI-SDS, and minimal lifetime BMI.

## Additional Information

**How to cite this article**: Geisler, D. *et al*. Increased anterior cingulate cortex response precedes behavioural adaptation in anorexia nervosa. *Sci. Rep.* **7**, 42066; doi: 10.1038/srep42066 (2017).

**Publisher's note:** Springer Nature remains neutral with regard to jurisdictional claims in published maps and institutional affiliations.

## Supplementary Material

Supplementary Information

## Figures and Tables

**Table 1:** Group characteristics.

	AN		HC			
	*Mean*	*SD*	*Mean*	*SD*	Test statistics	
**Demographic variables**					**T**	**p**
Age	16.0	2.6	16.3	2.6	−0.5	0.662
BMI	14.7	1.3	20.4	2.5	−12.0	<0.001
BMI SDS	−2.1	0.6	0.0	0.8	−11.7	<0.001
Minimal lifetime BMI	14.4	1.3	19.8	2.4	−11.7	<0.001
IQ	111.9	11.1	110.9	10.0	0.4	0.673
Handedness	0.5	2.0	1.7	3.7	−1.8	0.081
**Clinical variables**					**T**	**p**
EDI-2 total score	197.4	50.7	139.6	28.0	5.9	<0.001
EDI-2 perfectionism	19.6	6.0	15.7	4.2	3.3	0.002
BDI-II total score	19.5	11.6	5.5	5.7	6.5	<0.001
**Task relevant variables**					**F**	**p**
Total win [€]	5.0	1.8	5.2	2.5	0.2	0.655
Persistence	2.0	0.7	2.3	1.3	1.8	0.190
Lose-shift	30.3	6.6	27.0	7.4	4.6	0.035
Win-shift	5.9	5.5	6.3	5.3	0.1	0.780
Contingency reversal	9.0	1.8	8.8	1.9	0.3	0.561

Comparisons of demographic and clinical variables were examined using independent two-sample t-tests, differences in task relevant variables were examined using one-way ANCOVAs controlling for IQ. Means and standard deviations (SD) are given.

AN = anorexia nervosa patients; HC = healthy controls; BMI-SDS = body mass index standard deviation score; IQ = intelligence quotient; EDI-2 = Eating disorder inventory; BDI-II = Beck Depression Inventory; lose-shift = negative feedback that incurred a change in behaviour; win-shift = positive feedback that incurred a change in behaviour; *P*-values below 0.05 indicates a significant group difference; For more details on additional task performance measures see SI Table 2.1.
