# Supplementary material for: Increased anterior cingulate cortex response precedes behavioural adaptation in anorexia nervosa
Source: Sci Rep. 2017 Feb 13;7:42066. doi: 10.1038/srep42066 (PMC5304157; doi:10.1038/srep42066)
Supplement: Supplementary Information [file srep42066-s1.doc]

***Supplementary Information***

**Increased anterior cingulate cortex response precedes behavioural adaptation in anorexia nervosa**

Daniel Geisler MSc,^1,2^ Franziska Ritschel MSc, ^1,2^ Joseph A. King PhD, ^1,2^
Fabio Bernardoni PhD, ^1,2^ Maria Seidel MSc, ^1,2^ Ilka Boehm MSc, ^1,2^ Franziska Runge, ^1,2^
Thomas Goschke PhD,^3^ Veit Roessner MD,^2^ Michael N. Smolka MD,^4^ Stefan Ehrlich MD ^*,1,2^

^1^Division of Psychological and Social Medicine and Developmental Neuroscience,
Faculty of Medicine, Technische Universität Dresden,

Dresden, Germany

^2^Eating Disorder Treatment and Research Center, Department of Child and Adolescent Psychiatry, Faculty of Medicine, Technische Universität Dresden,

Dresden, Germany

^3^Department of Psychology, Institute of General Psychology, Biopsychology and Methods of Psychology, Technische Universität Dresden,

Dresden, Germany

^4^Department of Psychiatry and Neuroimaging, Technische Universität Dresden,

Dresden, Germany

# Supplementary Information (SI)

# Methods

## Participants

AN participants were recruited from specialised eating disorder programs of university child and adolescent psychiatry and psychosomatic medicine departments within 96 hours of initiating nutritional rehabilitation. Since nutritional rehabilitation has to be carried out with caution at the beginning of the treatment due to possible complications such as refeeding syndrome^1,2^ our patients had at that stage of treatment a calorie intake of about 1000 to 1500kcal per day.

BMI cut offs for AN patients were defined according to Kromeyer-Hauschild et al.^3^ and Hebebrand et al^4^.

HCs were recruited through advertisement among middle school, high school, and university students. For pairwise case-control age-matching an implementation of the Munkres algorithm was used^5^ resulting in a maximum difference of 1.7 years between the individuals within one pair.

HC participants were excluded prior to fMRI scanning if they had any history of psychiatric illness, a lifetime BMI below the 10^th^ age percentile (if younger than 18 years)/BMI below 18.5kg/m2 (if older than 18 years), or were currently obese (BMI not over 97th age percentile if younger than 18 years; BMI not over 30kg/m² if older than 18 years).

Participants of all study groups were excluded if they had a lifetime history of any of the following clinical diagnoses: organic brain syndrome, schizophrenia, substance dependence, psychosis NOS, bipolar disorder. Further exclusion criteria for all participants were IQ lower than 85; current inflammatory, neurologic or metabolic illness; chronic medical or neurological illness that could affect appetite, eating behaviour, or body weight (e.g. diabetes); clinical relevant anaemia; pregnancy; breast feeding.

## Psychiatric and psychological assessments

Exclusion criteria and possible confounding variables, e.g. the use of psychotropic medications and medical comorbidities, were obtained using the expert version of the Structured interview for anorexia and bulimia nervosa for DSM-IV (SIAB-Ex)^6^ and our own semi-structured interview. SIAB-EX is a well-validated 87-item semi-standardised interview that assesses the prevalence and severity of specific eating-related psychopathology over the past three months. The interview provides diagnoses according to the ICD-10 and DSM-IV and is suitable for adolescents as well as adults. It has been used widely in eating disorder research^7–9^. A good inter-rater reliability (k=.81) for the diagnostic interview has been demonstrated^10^. Interviews were conducted by clinically experienced and trained research assistants under the supervision of the attending child and adolescent psychiatrist.

For AN patients, comorbid psychiatric diagnoses other than eating disorders were derived from medical records and confirmed by an expert clinician with over 10 years of experience after careful chart review (including consideration of medical and psychiatric history, physical examination, routine blood tests, urine analysis, and a range of psychiatric screening instruments). All diagnostic information was ascertained at the time of treatment and the principal investigator of this study is also the chief consultant of the eating disorder treatment centre.

Psychiatric conditions in potential HC were ascertained using the same instruments as in patients. All interviews were carried out by trained graduate students (psychology or medicine). If there were any indications of psychiatric symptoms each case was discussed with a fully board-certified expert clinician and assessments were extended if necessary.

We used a short version of the German adaptation of the Wechsler Intelligence Scale for Children^11–13^ which included the following subtests: vocabulary, letter number sequencing, matrix reasoning, and symbol search. The short version of the German adaptation of the Wechsler Adult Intelligence Scale^14,15^ included the following subtests: picture completion, digit symbol coding, similarities and arithmetics.

The used BMI standard deviation score (BMI-SDS) provides an index of weight to height ratio that is corrected for age and gender^16,17^.

Handedness was assessed using a short version of the Annett Scale of Hand Preference^18^ as previously implemented in Gollub et al.^19^. This questionnaire asks for handedness in typical daily life situations as writing or brushing teeth. Response categories range from 0 ‘right hand’, 1 ‘both hands’ to 2 ‘left hand’. A mean score for handedness was calculated.

Study data were collected and managed using secure, web-based electronic data capture tools REDCap (Research Electronic Data Capture)^20^.

## Experimental Paradigm

Before entering the scanner, participants performed a training run to become acquainted with probabilistic errors. After a short instruction, they were confronted with the task as described above, but without any reversal of contingencies. The practice session was completed after 8 consecutive choices of the ‘correct’ stimulus or after a maximum of 30 trials.

To prevent confusion instructions were displayed again (after training) right before the scanner task started and participants were informed that the paradigm had been changed in a subtle way. Participants were instructed to maximize their gain and were informed that they will receive their total monetary win in addition to a fixed monetary compensation immediately after completing the experiments.

An adaptive mechanism was used to ensure that probabilistic feedback occurs in 20% of correct stimulus and 20% of incorrect stimulus choices for all subjects.

## Behavioural Data Analysis & Quality Control

To ensure data quality, we verified that no participant had a hit ratio (number of decisions for the ‘wrong’ figure divided by total number of trials) below or equal to 50% (performance below chance).

All statistical analysis of demographic and behavioural data were carried out using R^21^.

## MRI Data acquisition

Images were acquired using standard sequences with a 3T whole-body MRI scanner (TRIO; Siemens, Erlangen, Germany) equipped with a standard head coil.

The T1-weighted structural brain scans were acquired with rapid acquisition gradient echo (MP-RAGE) sequence with the following parameters: number of slices=176; repetition time=1900ms; echo time=2.26ms; flip angle (FA) of 9°; slice thickness of 1mm; voxel size of 1x1x1mm³; field-of-view (FoV) of 256 x 224mm²; bandwidth of 200 Hz/pixel.

The functional images were acquired by using a gradient-echo T2*-weighted echo planar imaging (EPI) with the following parameters: tilted 30° towards AC–PC line (to reduce signal dropout in orbitofrontal regions); number of volumes=656; number of slices=42; repetition time=2410ms; echo time=25ms; FA of 80°; 3mm in-plane resolution; slice thickness of 2 mm (1mm gap resulting in a voxel size of 3 x 3 x 2mm³); FoV of 192 x 192 mm²; bandwidth of 2112 Hz/pixel.

In the scanner, all stimuli were presented via a head-coil-mounted display system based on LCD technology (NordicNeuroLab AS, Bergen, Norway). Participants responded with two LUMItouch

keypads (Lightwave Medical Industries, Vancouver, Canada). Stimuli were presented using Presentation® software (v11.1, Neurobehavioral Systems, Berkeley, CA , USA).

## MRI Data Analysis

GLM_flex is a Matlab script that implements a standard partitioned error GLM that is different from the corrected pooled approach used in SPM8. Thus, GLM_flex is preferable for multifactorial repeated measures and testing of interactions since the standard partitioned error approach makes fewer assumptions with regard to equality of variance, independence, and the stability of covariance patterns across voxels.

The mask of the ventral striatum was specified by binarising a probabilistic map^22^ with a threshold value of 0.4. The vmPFC mask was created by merging the left and right frontal medial orbital cortex label from the Automated Anatomical Labelling (AAL) atlas provided in the Wake Forest University (WFU) PickAtlas for SPM^23,24^. For the lDLPFC and rDLPFC masks we merged the regions of superior and middle frontal gyrus in the WFU PickAtlas for the left and the right hemisphere, respectively, but excluded posterior parts with y<24 in MNI-space (as in our previous work^25,26^) The mask of the ACC was created by intersecting a sphere (centred at x=-2, y=22, z=34, radius=32mm) and a binarised probabilistic map (>0) from the Harvard-Oxford atlas^27^. The ROIs (SI Figure 1.1) were created using the AAL and the Harvard-Oxford atlases^24,27^.

All results were corrected based on permutations using 3DClustSim (<http://afni.nimh.nih.gov/pub/dist/doc/program_help/3dClustSim.html>) for each ROI (small volume correction, SVC). This tool runs Monte Carlo simulations to estimate the cluster size at which the false positive probability is below a given alpha level (α=.01) for a given voxelwise p-value level (p=.001). The simulations resulted in the following thresholds for minimal cluster size (k): for ACC k=97, for lDLPFC k=99, for rDLPFC k=97, for VS k=34, and for vmPFC k=32.

## Additional Analyses using Extracted BOLD data

To investigate the direction of changes in BOLD responses, the extracted beta values were subjected to a linear mixed model using the *lmer* procedure in the lme4 package in R^21,28,29^.

Predictors in the model included the fixed effects group, feedback, and their interaction, and a by-subject random adjustment for the intercept. The p-values for post-hoc tests were adjusted using the single-step method.

## Additional Analyses - PPI

On the first level, the physiological activity was obtained by calculating the first eigen-variate across the voxel within the seed region and adjusting for the effects of interest. For every participant a whole-brain GLM analysis was performed using the following regressors: the deconvolved physiological activity of the seed region, the three psychological factors (win, lose-stay, lose-shift), and the three products of the physiological activity and each psychological factor (referred as “PPI regressors”). On the second level, the estimates of the three PPI regressors were subjected to a whole brain group analysis using GLM_flex (see above). We conducted an F-test to explore the interaction of group and the PPI regressors. Due to the exploratory purpose of the PPI connectivity analysis we report results with a voxelwise threshold of p<0.001. We extracted the feedback specific beta values of the voxels belonging to clusters (voxelwise p<0.001, cluster extend of k>=30).

## Additional Analyses – Cortical Thickness

For measuring cortical thickness (CT) and grey matter volumes the structural data were registered, motion corrected, realigned, averaged and analysed in an automated manner with the FreeSurfer software suite (http://surfer.nmr.mgh.harvard.edu, version 5.1.0), a well-documented program for volumetric segmentation and cortical surface reconstruction^30–35^.

Using standard FreeSurfer procedures to measure CT, surface reconstruction was first performed for each hemisphere and included tessellation of the grey matter-white matter boundary, automated topology correction, and surface deformation following intensity gradients to optimally place the grey-white and grey-cerebrospinal fluid borders at the location where the greatest shift in intensity defines the transition to the other tissue class. The resulting surfaces were then used to calculate CT at each vertex as the closest distance from the grey-white boundary to the pial surface. The quality of the surface reconstruction and segmentation was assured according to FreeSurfer user guidelines by visual inspection of the resulting output and, if necessary, edited with minor manual intervention.

We extracted the CT values for the dACC cluster as follows: First, we created a surface-based ROI by mapping the volume of the dACC cluster on the surface of the FreeSurfer standard brain. Second, for every subject the surface-based ROI was mapped into native space and the average CT of all vertices within the ROI was extracted.

Associations between neural activity of the dACC cluster during lose-shift and CT were analysed in each group. Bonferroni correction was applied for the total number of tests.

# Results

## Additional Analyses – Cortical Thickness

The CT of the dACC cluster was reduced in AN vs. HC (AN: mean 2.9mm ± 0.2 SD; HC: mean 3.1mm ± 0.2 SD; t(67.6)=3.9, p<0.001). In the control group the extracted CT and the BOLD in the dACC during lose-shift were not correlated (r=-0.17, p=0.31). In the AN group, a trend towards a *positive* correlation was revealed (r=0.35, p=0.08). The fact that the trend was positive (and not negative as one would expect) weakens the speculation that our main finding might be an artifact of the brain atrophy in the patients.

**SI Table 2.1. Additional task performance measures compared between groups.** One-way ANCOVAs controlling for IQ were used. Means and standard deviations (SD) are given.

|  |  | ***AN*** | | ***HC*** | | ***test statistics*** | |
| --- | --- | --- | --- | --- | --- | --- | --- |
|  |  | *Mean* | *SD* | *Mean* | *SD* |  |  |
| Task relevant variables | | | | | | *F* | *p* |
| Lose |  | 47.5 | 5.4 | 46.8 | 6.1 | 0.3 | 0.61 |
| Win |  | 72.5 | 5.4 | 73.2 | 6.1 | 0.3 | 0.61 |
| Correct choices |  | 81.2 | 7.1 | 82.0 | 7.9 | 0.2 | 0.65 |
| RT after lose [ms] |  | 638.2 | 116.4 | 659.1 | 118.0 | 0.6 | 0.45 |
| RT after lose (stay) [ms] |  | 647.3 | 132.1 | 663.3 | 134.0 | 0.3 | 0.60 |
| RT after lose (shift) [ms] |  | 635.9 | 123.8 | 653.3 | 125.5 | 0.4 | 0.54 |
| RT after win [ms] |  | 629.9 | 112.6 | 640.2 | 114.2 | 0.2 | 0.69 |

AN=anorexia nervosa patients; HC=healthy controls; lose=occurrence of negative feedbacks; win=occurrence of positive feedbacks; Correct choices=number of trials in which participants chose the correct stimulus (independent of feedback type). P-values below .05 indicate a significant group difference.

**SI Table 2.2.** **Main effect of feedback.** Peak voxels of clusters within the a priori ROIs surviving FWE-correction.

| **ROI** | **Peak Voxel MNI Coordinates** | | | ***F*** |
| --- | --- | --- | --- | --- |
|  | x | y | z |  |
| *dACC* | -6 | 16 | 40 | 29.42 |
| *dACC* | 8 | 22 | 36 | 27.64 |
| *dACC* | 8 | 18 | 48 | 24.54 |
| *lDLPFC* | -28 | 32 | 36 | 12.64 |
| *lDLPFC* | -28 | 52 | 26 | 12.54 |
| *lDLPFC* | -42 | 44 | 24 | 11.31 |
| *rDLPFC* | 38 | 42 | 32 | 14.79 |
| *rDLPFC* | 50 | 30 | 32 | 11.72 |
| *rDLPFC* | 24 | 48 | 22 | 10.59 |
| *VS* | -10 | -4 | 2 | 22.86 |
| *VS* | 16 | 10 | 0 | 23.72 |

dACC=dorsal anterior cingulate cortex, lDLPFC=left dorso-lateral prefrontal cortex, rDLPFC=right dorso-lateral prefrontal cortex, VS=ventral striatum.

**
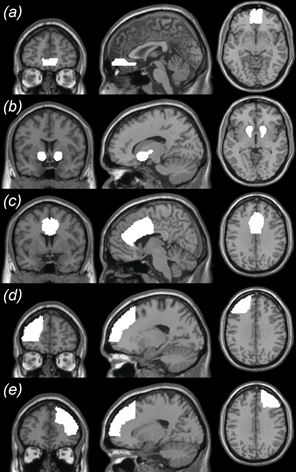
**

**SI Figure 1.1.** **Regions of interest.** (a) ventromedial prefrontal cortex - vmPFC, (b) ventral striatum - VS, (c) anterior cingulate cortex - ACC, (d) left dorsolateral prefrontal cortex – lDLPFC and (e) right dorsolateral prefrontal cortex – rDLPFC.


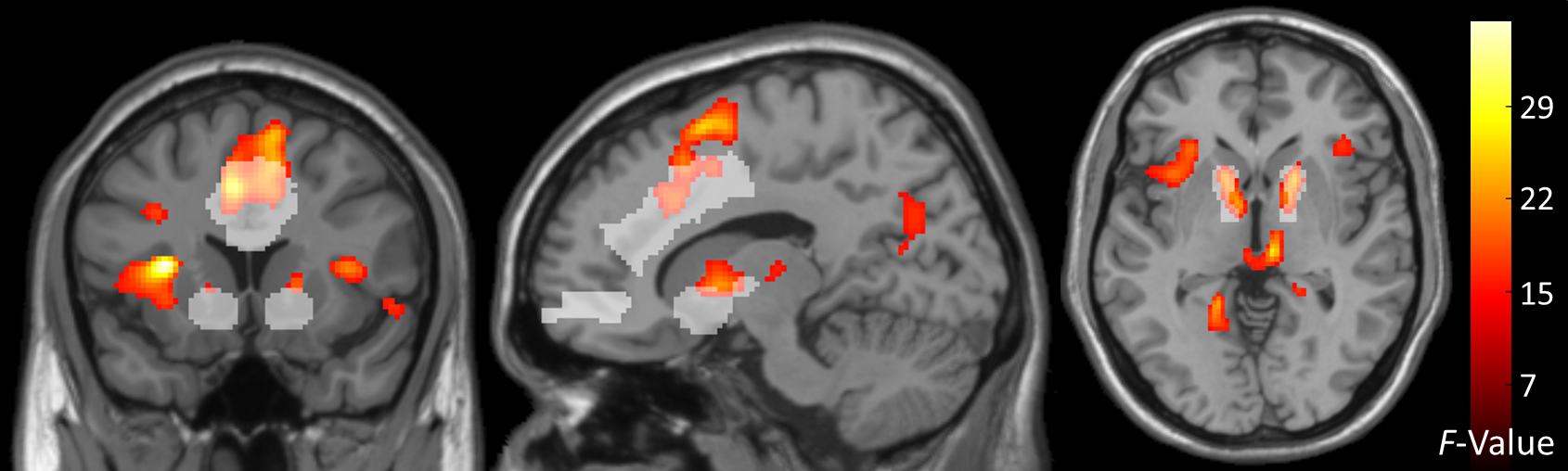


**SI Figure 2.1.** **Effects of Feedback.** Brain maps showing brain regions that are modulated by the feedback-condition (p_FWE_ < 0.05). The ROIs VS, vmPFC and ACC are highlighted in light grey.


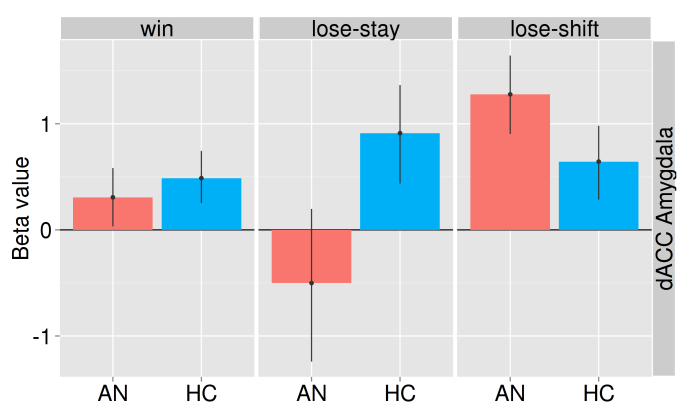


**SI Figure 2.2.** **Additional analysis of the amygdala cluster.** Identified during gPPI analysis based on beta value extraction with the MarsBaR toolbox for SPM^36^. Changes in functional connectivity of dACC and amygdala across groups and feedback conditions are displayed in bar charts.

# References

1. Ehrlich, S., Querfeld, U. & Pfeiffer, E. Refeeding oedema : an important complication in the treatment of anorexia nervosa. *Eur Child Adolesc Psychiatry* **15,** 241–243 (2006).

2. Tey, H. L., Lim, S. C. & Snodgrass, A. M. Refeeding oedema in anorexia nervosa. *Singapore Med J* **46,** 308–310 (2005).

3. Kromeyer-Hauschild, K. *et al.* Perzentile für den Body-mass-Index für das Kindes-und Jugendalter unter Heranziehung verschiedener deutscher Stichproben. *Monatsschrift Kinderheilkunde* **149,** 807–818 (2001).

4. Hebebrand, J., Casper, R., Treasure, J. & Schweiger, U. The need to revise the diagnostic criteria for anorexia nervosa. *J Neural Transm* **111,** 827–840 (2004).

5. Munkres, J. Algorithms for the Assignment and Transportation Problems. *Journal of the Society for Industrial and Applied Mathematics* **5,** 32–38 (1957).

6. Fichter, M. M. & Quadflieg, N. *Strukturiertes Inventar für Anorektische und Bulimische Essstörungen (SIAB). Fragebogen (SIAB-S) und Interview (SIAB-Ex) nach DSM-IV und ICD-10*. (Göttingen: Hogrefe, 1999).

7. Giel, K. E. *et al.* Attentional Processing of Food Pictures in Individuals with Anorexia Nervosa—An Eye-Tracking Study. *Biological Psychiatry* **69,** 661–667 (2011).

8. Kaye, W. H., Bulik, C. M., Thornton, L., Barbarich, N. & Masters, K. Comorbidity of Anxiety Disorders With Anorexia and Bulimia Nervosa. *AJP* **161,** 2215–2221 (2004).

9. Zipfel, S. *et al.* Focal psychodynamic therapy, cognitive behaviour therapy, and optimised treatment as usual in outpatients with anorexia nervosa (ANTOP study): randomised controlled trial. *Lancet* **383,** 127–137 (2014).

10. Fichter, M. & Quadflieg, N. The structured interview for anorexic and bulimic disorders for DSM-IV and ICD-10 (SIAB-EX): reliability and validity. *Eur. Psychiatry* **16,** 38–48 (2001).

11. Petermann, F. & Petermann, U. *Hamburg Wechsler Intelligenztest fuer Kinder IV (HAWIK-IV)*. (Huber, 2008).

12. Waldmann, H.-C. Kurzformen des HAWIK-IV: Statistische Bewertung in verschiedenen Anwendungsszenarien. *Diagnostica* **54,** 202–210 (2008).

13. Petermann, F. & Daseking, M. *Fallbuch HAWIK-IV.* (Hogrefe, 2009).

14. von Aster, M., Neubauer, A. C. & Horn, R. *WIE. Wechsler Intelligenztest für Erwachsene. Deutschsprachige Bearbeitung und Adaptation des WAIS–III von David Wechsler*. (Harcourt Test Services, 2006).

15. Donnell, A. J., Pliskin, N., Holdnack, J., Axelrod, B. & Randolph, C. Rapidly-administered short forms of the Wechsler Adult Intelligence Scale-3rd edition. *Arch Clin Neuropsychol* **22,** 917–924 (2007).

16. Hemmelmann, C., Brose, S., Vens, M., Hebebrand, J. & Ziegler, A. Perzentilen des Body-Mass-Index auch für 18- bis 80-Jährige? Daten der Nationalen Verzehrsstudie II. *DMW - Deutsche Medizinische Wochenschrift* **135,** 848–852 (2010).

17. Kromeyer-Hauschild, K. *et al.* Perzentile für den Body-mass-Index für das Kindes- und Jugendalter unter Heranziehung verschiedener deutscher Stichproben. *Monatsschr Kinderheilkd* **149,** 807–818 (2001).

18. Annett, M. A Classification of Hand Preference by Association Analysis. *British Journal of Psychology* **61,** 303–321 (1970).

19. Gollub, R. L. *et al.* The MCIC collection: a shared repository of multi-modal, multi-site brain image data from a clinical investigation of schizophrenia. *Neuroinformatics* **11,** 367–388 (2013).

20. Harris, P. A. *et al.* Research electronic data capture (REDCap) - A metadata-driven methodology and workflow process for providing translational research informatics support. **42(2),** 377–81 (2009).

21. R Core Team. *R: A Language and Environment for Statistical Computing*. (R Foundation for Statistical Computing, 2013).

22. Nielsen, F. ˚arup & Hansen, L. K. Automatic anatomical labeling of Talairach coordinates and generation of volumes of interest via the BrainMap database. in *Presented at the 8th International Conference on Functional Mapping of the Human Brain, June 2–6, 2002* (2002).

23. Maldjian, J. A., Laurienti, P. J., Kraft, R. A. & Burdette, J. H. An automated method for neuroanatomic and cytoarchitectonic atlas-based interrogation of fMRI data sets. *Neuroimage* **19,** 1233–1239 (2003).

24. Tzourio-Mazoyer, N. *et al.* Automated anatomical labeling of activations in SPM using a macroscopic anatomical parcellation of the MNI MRI single-subject brain. *Neuroimage* **15,** 273–289 (2002).

25. Ehrlich, S. *et al.* The COMT Val108/158Met polymorphism and medial temporal lobe volumetry in patients with schizophrenia and healthy adults. *Neuroimage* **53,** 992–1000 (2010).

26. Ehrlich, S. *et al.* Associations of cortical thickness and cognition in patients with schizophrenia and healthy controls. *Schizophr Bull* **38,** 1050–1062 (2012).

27. Desikan, R. S. *et al.* An automated labeling system for subdividing the human cerebral cortex on MRI scans into gyral based regions of interest. *Neuroimage* **31,** 968–980 (2006).

28. Bates, D., Maechler, M., Bolker, B. M. & Walker, S. *lme4: Linear mixed-effects models using Eigen and S4*. (2014).

29. Kuznetsova, A., Brockhoff, P. B. & Christensen, R. H. B. *lmerTest: Tests in Linear Mixed Effects Models*. (2014).

30. Desikan, R. S. *et al.* An automated labeling system for subdividing the human cerebral cortex on MRI scans into gyral based regions of interest. *Neuroimage* **31,** 968–80 (2006).

31. Fischl, B. *et al.* Whole brain segmentation: automated labeling of neuroanatomical structures in the human brain. *Neuron* **33,** 341–55 (2002).

32. Fischl, B. & Dale, A. M. Measuring the thickness of the human cerebral cortex from magnetic resonance images. *Proc. Natl. Acad. Sci. U.S.A.* **97,** 11050–11055 (2000).

33. Fischl, B., Sereno, M. I. & Dale, A. M. Cortical surface-based analysis. II: Inflation, flattening, and a surface-based coordinate system. *Neuroimage* **9,** 195–207 (1999).

34. Fischl, B., Sereno, M. I., Tootell, R. B. & Dale, A. M. High-resolution intersubject averaging and a coordinate system for the cortical surface. *Hum Brain Mapp* **8,** 272–84 (1999).

35. Dale, A. M., Fischl, B. & Sereno, M. I. Cortical surface-based analysis. I. Segmentation and surface reconstruction. *Neuroimage* **9,** 179–94 (1999).

36. Brett, M., Anton, J., Valabregue, R. & Poline, J. Region of interest analysis using an SPM toolbox. *NeuroImage* **16,** (2002).
